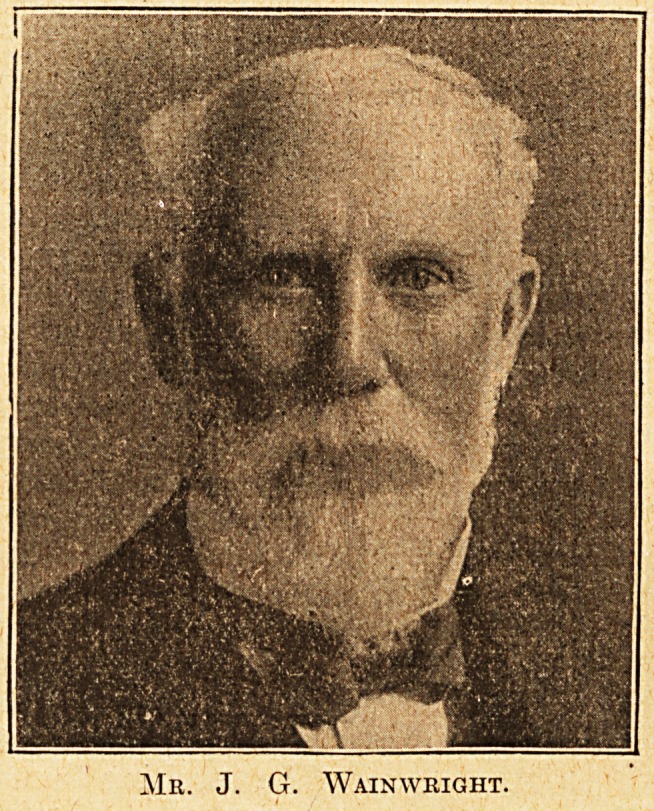# Resignation of Mr. J. G. Wainwright, the Treasurer

**Published:** 1917-06-23

**Authors:** 


### ST. THOMAS'S HOSPITAL.

### RESIGNATION OF MR. J. G. WAINWRIGHT, THE TREASURER.

At a Special Court of Governors of St. Thomas's Hospital held on Wednesday, June 20, 1917, the treasurer of St. Thomas's Hospital, Mr. J. G. Wainwright, laid down the reins of office and so closed his active career in the government of this hospital and the management of its affairs, which has been attended with extraordinary success.

For the past twenty-seven years (see THE HOSPITAL, January 14, 1911, pp. 479 *et seq.*), Mr. Wainwright has devoted the whole of his time and energies with marked success to the development and to foster the progress of this great hospital. On the death of Mr. Alderman Stone in March 1890 Mr. Wainwright was elected by a great majority to fill the office, for which he had shown an aptitude from the time he became a Governor of the hospital in November 1866. In July 1870 he was elected a member of the Audit Committee, and in April 1874 was duly elected one of the almoners, of which body he has remained a member ever since.

MR. J. G. WAINWRIGHT.

### ITS SCHEME OF GOVERNMENT.

By the Charter of Edward VI. the management of St. Thomas's Hospital was entrusted to the Mayor and Commonalty of the City of London and other Governors, and by the constitution of the hospital the active management is delegated to a Grand Committee of thirty members, of whom the treasurer is chairman. This body meets monthly, and is responsible for the conduct and management of the property and funds of the hospital. The executive work is largely carried out by the committee of four almoners who are elected at the June General Court from those Governors who have served or who are then serving on the Grand Committee. This body meets weekly, with the treasurer as chairman, and it is their duty to confer together upon all matters referred to them by the General Court or Grand Committee, and to join in making reports, if required, both in regard to the management of the property of the hospital and the administration of its internal affairs. How well and faithfully this work has been carried out by Mr. Wainwright and those Governors who have given him their cordial assistance and support in the Government of the hospital is shown by the high position to which the hospital has been raised and the cordial relations which exist between the Governors and the staff of the hospital.

### THE SHORTAGE OF FUNDS.

Though the present buildings were opened for use by Her Majesty Queen Victoria on June 21, 1871, funds had never up to the time of Mr. Wainwright's election as treasurer permitted the occupation of the whole of the building. When the railway company had decided to purchase the old site of the hospital in Southwark in 1862, a resolution was passed by the Governors at their Court in June of that year empowering the Grand Committee to secure accommodation for patients during the rebuilding of the hospital. It then appearing that all hope of arranging with the railway company was at an end, the Court empowered the Grand Committee to take all necessary measures for securing without delay accommodation for in-patients and out-patients in such place or places as the Committee may deem suitable, and to make arrangements for such of the officers and servants of the hospital as may be displaced from their residences in July and for the removal of the museum, library, etc. The Grand Committee was also empowered to take steps for erecting such buildings as may be required for the reception and accommodation of cases of accident, for urgent cases, and for dispensary business at a cost not exceeding £15,000.

### THE SELECTION OF A NEW SITE.

In October 1862 the Grand Committee considered a proposal to purchase the fee simple of about 15 acres of ground known as the Surrey Gardens. This site was temporarily occupied, and, the general feeling of the Governors being against purchase, it was rented for a term of five years. Various sites for the new hospital were considered, a proposal for the purchase of Bethlem Hospital being very seriously entertained, and the Governors of the two corporations met with a view to arranging, if possible, that the Governors of St. Thomas's Hospital should obtain the site on terms mutually advantageous. At the same time negotiations were entered into with the Metropolitan Board of Works for the purchase of a site on the proposed southern embankment of the river, and to report thereon to the Court of Governors.

### PLANS OF THE NEW HOSPITAL AND THE SITE PURCHASED.

The years 1863 and 1864 were years of serious discussion amongst the Governors, and it was not until January 1865 that the architect of the hospital was directed to prepare proper plans and designs for the new hospital and submit them to the Court of Governors. These plans and designs for the erection of a modern hospital on the site at Stangate, opposite the Houses of Parliament, as prepared by Mr. Henry Currey, were finally accepted in July 1865, and the first stone of the new hospital was laid on Wednesday, May 13, 1868, by Her Majesty Queen Victoria. The Governors then declared that the site had been chosen to combine the essential condition of an ample area with the convenience of ready access so urgently demanded by the public. The site was purchased for a sum of £100,000.

### The Buildings Justified by Experience.

The existing buildings are admirable proof of the wisdom of the Governors who were responsible for the acceptance of the plans prepared by Mr. Currey. Seven pavilions were placed at a distance of 125 feet apart, the centre court being increased to 200 feet, and thus ample sunlight and air were ensured to every part. External balconies were provided towards the river, whilst small wards were erected, contiguous to but not communicating with the general wards, for the reception of cases which it might be desirable to separate from other patients. No. 1 Block was devoted to the administration offices, and in the design for the new hospital provision was made for the accommodation of the Nightingale Home, as for the previous eight years nurses selected by Miss Nightingale had been trained, with great advantage to the public, in St. Thomas's Hospital. The museum, school buildings, lecture theatres, etc., were placed at the southern end of the ground, a position they still occupy. The total cost of the site, buildings, and fittings of the new hospital was estimated at a minimum of £500,000. Such were the buildings over which, with little or no alteration since their first erection, Mr. Wainwright was chosen to preside in 1890.

### Figures for 1890 and 1916 Compared.

At that time the expenditure on the upkeep of the hospital was £40,542, of which £6,680 went in provisions, £4,651 in surgery and dispensary, £15,331 on salaries to officers, nurses, and servants, of which the sisters' and nurses' salaries amounted to £3,152. It is interesting to contrast these figures with the expenditure in 1916, when provisions cost £35,624; surgery and dispensary, £7,823; salaries and wages, £29,337, of which the salaries of sisters and nurses amounted to £5,508, and a total ordinary expenditure of £105,560.

### The Treasurer, the Medical Staff and School.

Mr. Wainwright must indeed be proud of the noble monument to his successful and diligent work which he now leaves to the trusteeship of his successor. At the time of Mr. Wainwright's election there was little or no co-operation between the Governors and the medical staff, but by a judicious handling of difficult questions as they have arisen, and by calling the staff to his councils, Mr. Wainwright has secured not only their cordial help and co-operation, but their admiration and appreciation of his successful administration of St. Thomas's Hospital. In November 1892 the erection of extensions to the medical school buildings was decided upon, and a sum of £16,000 was expended upon them.

### Paying Patients and 100 Additional Beds Provided.

In 1878 it was resolved that two of the vacant wards should be appropriated for the reception of paying patients, but there still remained empty wards containing 100 beds, which might be opened and relief given which was urgently needed. This was a state of affairs which a man of the energy of Mr. Wainwright could not expect to endure patiently, and accordingly in November 1894, encouraged by the President, H.R.H. the Duke of Connaught, it was determined to make an appeal to the public for funds to discharge the balance of the debt incurred at the time of the rebuilding of the hospital and to secure funds for the opening of the empty wards. The Lord Mayor concurred, and a meeting was held at the Mansion House on February 13, 1895, and the needs of the hospital were laid before the public. Alderman Sir Stuart Knill, Bart., presided in the absence of the Lord Mayor. The Livery Companies of London nobly responded to the appeal, and from that date began the complete reorganisation of the hospital. In January 1898 the Mercers' Company voted a sum of £10,000, as a gift in commemoration of the sixtieth year of the reign of Her Majesty the Queen, for the endowment of a certain number of beds to be named after the company, and these are all placed in what is known as City Ward. At that time the expenditure on the general upkeep of the hospital had risen to £44,259. In January 1896 two of the closed wards had been opened, and in March 1899 the Court adopted plans which had been prepared for building new operating theatres and new children's wards. The resulting theatres and wards have never been surpassed. The building of the new casualty department was decided on.

### The Late Charles Gassiot's Munificence.

In 1902 the hospital benefited by the beneficent bequest of Mr. Charles Gassiot, a Governor of the hospital and a life-long friend of Mr. Wainwright, who bequeathed his residuary estate to the hospital, subject to his wife's interest in a portion thereof. In consequence, in November of that year it was resolved to proceed with the rebuilding of Block 1 and the erection of a Nurses' Home. At the same time accommodation was provided on two floors of the new building for paying patients, and the wards thus set free were devoted to the reception of ordinary hospital patients. From this time forward the buildings of the hospital were fully occupied. The erection of the Nurses' Home was of material assistance to Mr. Wainwright in his successful efforts to bring the standard of nursing in the Nightingale Training School to its present high state of perfection.

*For the conclusion of this article see page 230.*

### St. Thomas's Hospital (*continued from page* 228).

### An Eventful Decade.

During the past decade, the greatest progress in medical and surgical work has been made in London. The Governors and staff of St. Thomas's Hospital, led by their treasurer, have nobly responded to the call. It follows, when Mr. Wainwright, full of years and honour, relinquishes his life's work, he may well and truly say that great changes have been carried out, for the whole hospital has been reorganised.

### Youth at its Zenith.

Finally, mention must be made of the Louis Jenner Laboratory and the invaluable pathological work carried out there, to the tuberculosis dispensary, to the maternity wards, to the massage and medical electricity department, to the $x$-ray department, to the lady almoner's department which is magnificently administered, and last, but not least, to the new venereal department, to complete the evidence and prove to demonstration, that this ancient institution has answered the calls made upon it with a surprising vigour worthy of youth at its zenith.

### An Opportunity for the Governors and the Nation.

Who will not sympathise with Mr. Wainwright in the one regret to which he gave expression to the Court on Wednesday, 20th inst.? The hospital has but one want not supplied, and that is a new and efficient out-patient department. Had not this terrible war occurred, the treasurer had resolved that by now such a department should be well in hand. In his farewell to the Governors he expressed the hope that as soon as peace is secured every effort will be made to supply this need, and thus complete the organisation of St. Thomas's Hospital, which deserves well of the nation and all residents in the Metropolis of the Empire.

## Figures and Tables

**Figure f1:**